# Evaluation of eczema, asthma, allergic rhinitis and allergies among the grade-7 children of Iqaluit

**DOI:** 10.1186/s13223-019-0341-6

**Published:** 2019-04-23

**Authors:** Ahmed Ahmed, Allan Becker

**Affiliations:** 1 Department of Pediatrics, University of Ottawa, Ottawa, ON Canada; 2 Section of Allergy and Clinical Immunology, Department of Pediatrics and Child Health, University of Manitoba, Winnipeg, MB Canada

**Keywords:** Asthma, Allergic rhinitis, Eczema, Allergies, Inuit, Nunavut, Subarctic, Children

## Abstract

**Background:**

Little is known about the prevalence of asthma, allergic rhinitis, eczema and allergies among Canadian Inuit children, especially those living in the arctic and subarctic areas.

**Methods:**

A cross-sectional study among grade seven students attending schools in Iqaluit, the capital city of Nunavut, was conducted during the 2016/2017 school year. We used the International Study of Allergy and Asthma in Children (ISAAC) questionnaire with added questions relevant to the population. In addition, skin prick tests (SPT) were conducted to test for sensitization to common food and environmental allergens.

**Results:**

The prevalence of current asthma is 5.2%, all of them were males and 2/3 of them were Inuit and all had a previous respiratory hospitalization. Past asthma prevalence is 8.6%, 60% males and 60% Inuit. There was an inverse relationship to crowdedness possibly as a confounding factor because of getting a higher prevalence among the non-Inuit who usually live in less crowded houses. Current allergic rhinitis prevalence is 8.6%, 60% of the cases were among the mixed Inuit/Caucasian ethnicity while no cases among the non-Inuit, there was a female predominance 3:2. Past history of allergic rhinitis prevalence is 10.3%, half of the cases were among the mixed ethnicity (5.2% of that ethnicity) followed by Inuit (3.4%) and non-Inuit (1.7%), female: Male ratio 1:1. Current eczema prevalence was 27.6%, with half of the cases among the mixed ethnicity (13.8% of that group), followed by Inuit (8.6%). There was a female predominance with protective effect of exclusive breastfeeding. Past eczema prevalence 34.5%, with half of the cases were among the mixed ethnicity (17.2% of that group), followed by Inuit (10.3%). There was a female predominance. We noted a high rate of sensitization to Cat at 29.2%, most of the cases were among the mixed ethnicity, while absent sensitization to other common inhalant allergens.

**Conclusion:**

While being cautious about firm conclusions due to the small sample size and power, the noticed variations in the prevalence and risk factors of asthma, allergic rhinitis and eczema among different ethnicities living at the same subarctic environment might be related to several possible explanations like genetic, gene-environment interaction and/or lifestyle factors, it was out of the scope of this study to determine the causality of such variation in prevalence, which emphasizes the need for further investigation.

## Background

Little is known about the prevalence of asthma, allergic rhinitis, eczema and allergies among the Canadian Inuit children, especially those living in the Canadian territory of Nunavut. This is the first study addressing that issue among adolescents in Nunavut that was part of a broader study that included 6–7 year old children [1]. Improving our knowledge of those conditions among the Inuit children carries the potential of improving their prevention and management.

Nunavut is a sparsely populated area of arctic and subarctic tundra located above latitude 60° with a population of 38,456 as of April 1st, 2018, About 84 percent of the residents claim ancestry from native tribes; the largest of these Nunavut tribes is the Inuit (83%), formerly known as the Eskimos (a derogatory term meaning “eaters of raw meat”).

Huge areas of its surface are sheathed in ice year-round, having more than 50% of Nunavut’s landmass above the Arctic Circle with not a single tree in the entire area. Iqaluit, is the largest community in Nunavut and its capital city, with a population of 7590 (2016). Iqaluit encompasses an area of about 52 km^2^ and has the highest population of Inuit (4208) of all Canadian cities over 5000 people at 55.4%. Winters can be very harsh with average temperatures of − 27 °C in Iqaluit which limits the time of out-door activities for most of the year [2–4].

The International Study of Asthma and Allergies in Childhood (ISAAC) has shown clear differences in the prevalence of atopic diseases between different countries, geographic areas and within the same countries over time [5, 6]. A number of studies have also reported a higher prevalence of allergy and eczema in colder, more northern regions [7–11].

There is a wide variation in the prevalence of allergic conditions among Canadian adolescents aged 13–14 years that ranged from 13.7 to 33.0% for current asthma, 14.6–22.6% for allergic rhinoconjunctivitis and 8.2–10.4% for atopic eczema [12].

The ISAAC study has never been conducted in Nunavut. Forsey found an eczema rate of 16.5% among Inuit children (age 2–12 years) in the Labrador area (a region in the Canadian province of Newfoundland and Labrador south eastern to Nunavut with an Inuit minority). Two-thirds of those children presented with moderate or severe eczema, with a female/male ratio of 2:1 [13].

Asthma and allergies are among the most common chronic conditions reported by parents/guardians of indigenous children above the age of 12 years at 8.8% and 13.5% respectively [14]. Chang et al. found that children and adults with Inuit ancestry, but living outside Nunavut and off the First Nations reserves in other provinces, had a significantly lower prevalence of asthma and allergies compared to children from other Indigenous groups [15].

Hansen et al. reported an increasing prevalence of asthma ever and AR ever among schoolchildren (7–14 years) in Norway, together with a considerably increase in current asthma, AR and eczema between 1995 and 2008 [11], this emphasizes the need for a baseline information to compare to in the future.

In our study among grade one children, none had sensitized to house dust mite [1] and in a study of Inuit school children in Northern Quebec, specific sensitization to dust mite was very unusual with almost complete absence of mite allergen in house dust [16]. Alaskan native children residing in rural Alaska also have a low prevalence of allergic sensitization to common inhalant allergens [17].

## Materials and methods

The city of Iqaluit was chosen because it is the most populated city in Nunavut. The study was approved by the Research Ethics Board at the University of Manitoba and received permission from the Nunavut Research Institute.

### Study design

The study was conducted between December 2016 and February 2017 at the Qikiqtani General Hospital (Iqaluit). It is a cross-sectional with the study population being all grade seven students attending the only intermediate school at Iqaluit during the academic year 2016/2017. There were 108 students in grade 7, and we had no exclusion criteria. The families were contacted by phone multiple times over 2 months by the study assistants. The study included two components; a questionnaire and skin prick testing.

### The questionnaire

A questionnaire of 30 questions, was adopted with modification from the ISAAC study with additional questions relevant to the Nunavut population including locally applicable risk factors. For a detailed description please refer to our previous publication [1]. The consent form and assent form were available in English and Inuktitut languages, all the parents were satisfied to use the English version. Two study coordinators were hired, including a local Inuit fluent in Inuktitut, to minimize the language barrier bias, however, all participants chose to fill the English form.

### Skin testing

The skin prick testing by the epicutaneous technique to 14 common allergens (food and inhalant) was performed and interpreted by Dr. Ahmed Ahmed at the Qikiqtani General Hospital outpatient clinic over 2 days in February 2017. The allergen extracts, the testing devices and the procedures have been described in our previous article describing the findings among grade one students [1].

### Data analysis

The statistical analyses was performed using IBM SPSS v.22 (BM Corp. Released 2013. IBM SPSS Statistics for Windows, Version 22.0. Armonk, NY: IBM Corp.)

Data were analyzed using correlations, cross tabulations (Chi-square), and cross tabulations with risk analyses (odds ratios, 95% confidence interval) and used the G power that relies on the effect size, the sample and the degree of freedom to calculate a post hoc power number.

## Results

Of the 108 grade seven students in Iqaluit at the time of the study 58 families (53.7%) provided consent for the child to be enrolled in the study (all agreed to participate in both parts of the study; the questionnaire and the skin prick test) but only 24 children (22.2% of the total cohort) attended the skin prick testing despite a reminder call a day earlier.

### Study demographics

The ethnic distribution of participants who completed the questionnaire (58 cases) was as follows: Inuit 34/58 (58.6%), non-Inuit 6/58 (10.3%) and mixed ethnicity (one of parent being Inuit) 18/58 (31%). There were 30 females and 28 males. Of those who underwent skin testing (24 children; 12 each male and female), 12 (50%) were Inuit, 3 (12.5%) non-Inuit and 9 (37.5%) of mixed ethnicity.

### Asthma prevalence

Following the standard ISAAC approach, based on the ISAAC questionnaire primary questions, the current asthma prevalence was 5.2%, all were males, 2/3 were Inuit and none were of the mixed ethnicity. When looking on the prevalence among each ethnicity, the highest prevalence of current asthma was among the non-Inuit at 16.7% compared to 5.8% among the Inuit. The data suggested relationship to previous respiratory hospitalization (all were previously hospitalized), No significant relation was found to smoking exposure, dog ownership, a history of exclusive breast feeding, being ever outside Nunavut, family history of asthma or TB vaccination.

The past asthma prevalence was 8.6%, with 60% males and 60% Inuit, but none were of mixed ethnicity. When considering the prevalence among each group, the highest prevalence of past asthma is noticed among the non-Inuit at 33.3% compared to 8.8% among the Inuit. Again the data suggested a relationship to previous respiratory hospitalization (OR 14.4, CI 1.93–107.72). No significant relation was found to smoking exposure, dog ownership, exclusive breast feeding, being ever outside Nunavut, family history of asthma or TB vaccination (Table 1).

**Table 1:** Summary of the findings in relation to current and past asthma

The number of students with filled questionnaires: 58 out of 108	Current asthma (n = 3)	Past asthma (n = 5)
Male: female	3:0	3:2
Odds ratio	n/a	1.68
95% CI	n/a	.26–10.89
Ethnicity		
Inuit n (%)	2 (3.4)	3 (5.2)
Mixed n (%)	0 (.0)	0 (.0)
Non-Inuit n (%)	1 (1.7)	2 (3.4)
Total n (%)	3 (5.2)	5 (8.6)
Chi-square (p-value)	2.64 (.191)	5.12 (.051)
Smoker (yes: no)	2:1	3:2
Odds ratio	.683	.488
95% CI	.057–8.12	.073–3.25
Crowdedness correlation (p)	.114 (.40)	− .025 (.86)
Cat owner (yes: no)	1:2	1:4
Odds ratio	27.0	13.0
95% CI	1.20–605.63	.679–249.0
Dog owner (yes: no)	0:3	3:2
Odds ratio	n/a	1.81
95% CI	n/a	.280–11.75
Any pet owner (yes: no)	1:2	4:1
Odds ratio	.387	3.57
95% CI	.033–4.53	.374–34.11
Exclusive breast feeding (yes: no)	1:2	1:4
Odds ratio	.371	.169
95% CI	.032–4.34	.018–1.623
Being ever outside Nunavut (yes: no)	2:1	4:1
Odds ratio	.40	.84
95% CI	.03–4.90	.083–8.40
Previous respiratory hospitalization (yes: no)	3:0	3:2
Odds ratio	n/a	14.4
95% CI	n/a	1.93–107.72
TB vaccination (yes: no)	2:0	3:0
Odds ratio	n/a	n/a
95% CI	n/a	n/a
Family history of food allergy (yes: no)	0:3	1:4
Odds ratio	n/a	2.15
95% CI	n/a	.20–23.21
Family history of environmental allergy (yes: no)	0:3	1:3
Odds ratio	n/a	1.03
95% CI	n/a	.98–10.83
Family history of asthma (yes: no)	0:3	1:4
Odds ratio	n/a	1.28
95% CI	n/a	.126–13.02
Family history of eczema (yes: no)	0:3	2:3
Odds ratio	n/a	2.60
95% CI	n/a	.381–17.72

*CI* confidence interval

### Prevalence of allergic rhinitis

Current allergic rhinitis prevalence was 8.6%, 60% of the cases are among the mixed ethnicity (16.7% of that ethnicity), followed by Inuit (5.8%) while no cases among the non-Inuit. Female predominance 3:2. No significant relation was found to pet ownership, smoking exposure, exclusive breast feeding or being ever outside Nunavut.

Past allergic rhinitis prevalence was 10.3%, half of the cases were among the mixed ethnicity (16.7% of that ethnicity), followed by non-Inuit (16.7%) and Inuit (5.8%). Female: Male ratio 1:1. No significant relation was found to pet ownership, smoking exposure, exclusive breast feeding or being ever outside Nunavut (Table 2).

**Table 2:** Summary of the findings in relation to current and past allergic rhinitis

The number of students with filled questionnaires: 58 out of 108	Current allergic rhinitis (n = 5)	Past allergic rhinitis (n = 6)
Male: female	2:3	1:1
Odds ratio	.69	1.08
95% CI	.11–4.49	.20–5.85
Ethnicity		
Inuit n (%)	2 (3.4)	2 (3.4)
Mixed n (%)	3 (5.2)	3 (5.2)
Non-Inuit n (%)	0 (.0)	1 (1.7)
Total n (%)	5 (8.6)	6 (10.3)
Chi-square (p-value)	1.87 (.37)	2.31 (.32)
Smoker (yes: no)	4:1	2:1
Odds ratio	1.44	.67
95% CI	.15–14.0	.11–4.07
Crowdedness correlation (p)	− .118 (.38)	− .09 (.48)
Cat owner (yes: no)	0:5	0:6
Odds ratio	n/a	n/a
95% CI	n/a	n/a
Dog owner (yes: no)	4:1	5:1
Odds ratio	5.22	6.82
95% CI	.55–49.88	.74–62.55
Any pet owner (yes: no)	4:1	5:1
Odds ratio	3.57	4.63
95% CI	.37–34.11	.51–42.41
Exclusive breast feeding (yes: no)	3:2	2:1
Odds ratio	1.19	1.64
95% CI	.18–7.73	.28–9.79
Being ever outside Nunavut (yes: no)	4:1	5:1
Odds ratio	.84	1.07
95% CI	.08–8.40	.11–10.31
Previous respiratory hospitalization (yes: no)	2:3	1:2
Odds ratio	5.22	3.83
95% CI	.72–37.85	.57–25.60
TB vaccination (yes: no)	5:0	5:1
Odds ratio	n/a	n/a
95% CI	n/a	n/a
Family history of food allergy (yes: no)	1:4	1:4
Odds ratio	2.15	2.15
95% CI	.20–23.21	.20–23.21
Family history of environmental allergy (yes: no)	1:1	2:3
Odds ratio	3.46	2.24
95% CI	.44–27.42	.33–15.17
Family history of asthma (yes: no)	1:4	1:5
Odds ratio	1.28	1.00
95% CI	.13–13.02	.10–9.75
Family history of eczema (yes: no)	2:3	1:1
Odds ratio	2.60	4.33
95% CI	.38–17.72	.75–25.11

*CI* confidence interval

### Prevalence of eczema

The prevalence of current eczema was 27.6%, half of the cases were among the mixed ethnicity (44.4% of that ethnicity), still highest prevalence among the non-Inuit ethnicity at 50% while only 14.7% among the Inuit. There was a protective effect of exclusive breastfeeding (p .84), No significant relation was found to pet ownership, being ever outside Nunavut, family history of eczema or TB vaccination.

Past eczema prevalence was 34.5%, half of the cases were among the mixed ethnicity (55.6% of that ethnicity), with highest prevalence among the non-Inuit at 66.7% while 17.6% among the Inuit. Female predominance. There was a protective effect of exclusive breastfeeding (OR .16, CI .04–.57), but no significant relation was found to pet ownership, being ever outside Nunavut, family history of eczema or TB vaccination (Table 3).

**Table 3:** Summary of the findings in relation to current and past eczema

The number of students with filled questionnaires: 58 out of 108	Current eczema (n = 16)	Past eczema (n = 20)
Male: female	5:11	3:7
Odds ratio	.38	.31
95% CI	.11–1.27	.10–.99
Ethnicity		
Inuit n (%)	5 (8.6)	6 (10.3)
Mixed n (%)	8 (13.8)	10 (17.2)
Non-Inuit n (%)	3 (5.2)	4 (6.9)
Total n (%)	16 (27.6)	20 (34.5)
Chi-square (p-value)	6.99 (.03)	10.44 (.01)
Smoker (yes: no)	1:1	9:11
Odds ratio	.20	.10
95% CI	.06–.71	.03–.38
Crowdedness correlation (p)	− .143 (.29)	− .176 (.19)
Cat owner (yes: no)	1:15	1:19
Odds ratio	2.73	1.95
95% CI	.16–46.51	.12–32.88
Dog owner (yes: no)	3:5	1:1
Odds ratio	.60	1.24
95% CI	.19–1.95	.42–3.66
Any pet owner (yes: no)	7:9	11:9
Odds ratio	.53	.99
95% CI	.17–1.69	.33–2.94
Exclusive breast feeding (yes: no)	1:3	7:13
Odds ratio	.16	.26
95% CI	.04–.57	.08–.82
Being ever outside Nunavut (yes: no)	13:2	17:2
Odds ratio	1.53	2.27
95% CI	.29–8.17	.43–11.92
Previous respiratory hospitalization (yes:no)	1:15	1:9
Odds ratio	.33	.59
95% CI	.04–2.95	.11–3.25
TB vaccination (yes: no)	14:0	17:1
Odds ratio	n/a	n/a
95% CI	n/a	n/a
Family history of food allergy (yes: no)	1:15	3:16
Odds ratio	.43	1.94
95% CI	.05–3.98	.35–10.72
Family history of environmental allergy (yes: no)	3:11	2:7
Odds ratio	.79	.83
95% CI	.18–3.42	.22–3.17
Family history of asthma (yes: no)	1:7	3:17
Odds ratio	.63	.82
95% CI	.12–3.44	.18–3.73
Family history of eczema (yes: no)	4:11	8:11
Odds ratio	1.41	5.64
95% CI	.35–5.62	1.41–22.48

*CI* confidence interval

### Prevalence of reported food and environmental allergy

The reported history of food allergy was 3.4%, all among females and all of mixed ethnicity (11.1% of that ethnicity). The skin prick testing to common food allergens showed only one female child of the mixed ethnicity was sensitized to peanut, tree nut and soy.

The reported allergy to medications was 5.2%, with an equal number of cases among all groups with the highest prevalence among the non-Inuit at 16.7% followed by the mixed ethnicity at 5.6% but only 2.9% among the Inuit.

The reported history of anaphylaxis from any cause was 5.2%, all among Inuit males (8.8% of all Inuit) (Table 4).

**Table 4:** Summary of the findings in relation to reported history of food and environmental allergies

The number of students with filled questionnaires: 58 out of 108	Reported food allergy (n = 2)	Reported med allergy (n = 3)	Reported anaphylaxis (n = 3)
Male: female	0:2	2:1	3:0
Odds ratio	n/a	2.23	n/a
95% CI	n/a	.19–26.06	n/a
Ethnicity			
Inuit n (%)	0 (.0)	1 (1.7)	3 (5.2)
Mixed n (%)	2 (3.4)	1 (1.7)	0 (.0)
Non-Inuit n (%)	0 (.0)	1 (1.7)	0 (.0)
Total n (%)	2 (3.4)	3 (5.2)	3 (5.2)
Chi-square (p-value)	3.74 (.17)	2.46 (.31)	1.49 (.67)
Smoker(s) at home (yes: no)	2:0	2:1	2:1
Odds ratio	n/a	.68	.68
95% CI	n/a	.06–8.12	.06–8.12
Crowdedness correlation (p)	.184 (.17)	− .04 (.79)	− .11 (.41)
Cat ownership (yes: no)	0:2	0:3	0:3
Odds ratio	n/a	n/a	n/a
95% CI	n/a	n/a	n/a
Dog ownership (yes: no)	1:1	2:1	2:1
Odds ratio	1.15	2.4	2.4
95% CI	.07–19.38	.21–28.05	.21–28.05
Any pet owner (yes: no)	1:1	2:1	2:1
Odds ratio	.81	1.67	1.67
95% CI	.05–13.55	.14–19.48	.14–19.48
Exclusive breast feeding (yes: no)	1:1	3:0	2:1
Odds ratio	.78	n/a	1.60
95% CI	.05–13.02	n/a	.14–18.72
Being ever outside Nunavut (yes: no)	1:1	3:0	3:0
Odds ratio	.20	n/a	n/a
95% CI	.01–3.43	n/a	n/a
Previous respiratory hospitalization (yes: no)	0:2	1:2	1:2
Odds ratio	n/a	3.43	3.43
95% CI	n/a	.27–42.96	.28–42.96
TB vaccination (yes: no)	2:0	2:1	3:0
Odds ratio	n/a	n/a	n/a
95% CI	n/a	n/a	n/a
Family history of food allergy (yes: no)	0:2	0:2	0:3
Odds ratio	n/a	n/a	n/a
95% CI	n/a	n/a	n/a
Family history of environmental allergy (yes: no)	1:1	0:3	1:2
Odds ratio	3.25	n/a	1.58
95% CI	.19–55.98	n/a	.13–19.03
Family history of asthma (yes: no)	1:1	0:3	0:3
Odds ratio	5.5	n/a	n/a
95% CI	.31–97.23	n/a	n/a
Family history of eczema (yes: no)	0:2	2:1	0:3
Odds ratio	n/a	8.2	n/a
95% CI	n/a	.67–99.7	n/a

*CI* confidence interval

### Prevalence of sensitization to the tested environmental allergens

The prevalence of sensitization to tree pollen was 8.3%, all of them of the mixed ethnicity (22.2% of that group).

The prevalence of sensitization to grass pollen 20.8%, most of the cases were among the mixed ethnicity with the prevalence among that group of 33.3%, while 8.3% among the Inuit. Male to female ratio 4:1.

The prevalence of sensitization to cat was 29.2%, most of the cases were among the mixed ethnicity with the prevalence among that group of 44.4% while 33.3% among the non-Inuit and 16.7% among the Inuit ethnicity. Male to female ratio 3:4. (Table 5).

**Table 5:** Summary of the findings in relation to positive skin prick tests (Environmental)

The number of students underwent skin prick test: 24 out of 108	Positive skin prick test trees (n = 2)	Positive skin prick test grass (n = 5)	Positive skin prick test cat (n = 7)
Male: female	1:1	4:1	3:4
Odds ratio	1.00	5.5	.67
95% CI	.06–18.09	.51–59.01	.11–3.93
Ethnicity			
Inuit n (%)	0 (.0)	1 (4.2)	2 (8.3)
Mixed n (%)	2 (8.3)	3 (12.5)	4 (16.7)
Non-Inuit n (%)	0 (.0)	1 (4.2)	1 (4.2)
Total n (%)	2 (8.3)	5 (20.8)	7 (29.2)
Chi-square (p-value)	2.94 (.37)	2.61 (.27)	2.14 (.40)
Smoker(s) at home (yes: no)	2:0	4:1	6:1
Odds ratio	n/a	1.43	2.50
95% CI	n/a	.13–16.03	.24–26.48
Crowdedness correlation (p)	.402 (.052)	.396 (.06)	.244 (.25)
Cat ownership (yes: no)	0:2	1:4	0:7
Odds ratio	n/a	n/a	n/a
95% CI	n/a	n/a	n/a
Any pet owner (yes: no)	2:0	3:2	4:3
Odds ratio	n/a	.54	.41
95% CI	n/a	.07–4.20	.06–2.66
Exclusive breast feeding (yes: no)	2:0	3:2	4:3
Odds ratio	n/a	.69	.56
95% CI	n/a	.09–5.29	.09–3.45
Being ever outside Nunavut (yes: no)	1:1	4:1	7:0
Odds ratio	.05	.22	n/a
95% CI	.002–1.46	.01–4.36	n/a
Previous respiratory hospitalization (yes:no)	0:2	2:3	1:6
Odds ratio	n/a	3.56	.54
95% CI	n/a	.41–31.23	.05–5.94
TB vaccination (yes: no)	n/a	n/a	n/a
Odds ratio	n/a	n/a	n/a
95% CI	n/a	n/a	n/a
Family history of food allergy (yes: no)	1:1	1:4	1:6
Odds ratio	6.0	1.25	.72
95% CI	.29–124.1	.10–15.5	.06–8.46
Family history of environmental allergy (yes: no)	2:0	2:3	2:5
Odds ratio	n/a	1.33	.67
95% CI	n/a	.17–10.25	.10–4.58
Family history of asthma (yes: no)	2:0	2:3	1:6
Odds ratio	n/a	3.33	.50
95% CI	n/a	.38–29.39	.05–5.51
Family history of eczema (yes: no)	0:2	0:5	3:4
Odds ratio	n/a	n/a	1.25
95% CI	n/a	n/a	.21–7.62
Having current asthma (yes: no)	0:2	1:4	0:7
Odds ratio	n/a	n/a	n/a
95% CI	n/a	n/a	n/a
Having past asthma (yes: no)	0:2	1:4	1:6
Odds ratio	n/a	1.33	.78
95% CI	n/a	.11–16.48	.07–9.08
Having current eczema (yes: no)	0:2	2:3	2:5
Odds ratio	n/a	1.87	.96
95% CI	n/a	.24–14.65	.14–6.70
Having past eczema (yes: no)	0:2	2:3	2:5
Odds ratio	n/a	1.14	.57
95% CI	n/a	.15–8.59	.09–3.83
Having current allergic rhinitis (yes: no)	0:2	0:5	0:7
Odds ratio	n/a	n/a	n/a
95% CI	n/a	n/a	n/a
Having past allergic rhinitis (yes: no)	0:2	0:5	0:7
Odds ratio	n/a	n/a	n/a
95% CI	n/a	n/a	n/a

*n/a* not applicable, *CI* confidence interval

## Discussion

Our study provides the first data on the prevalence of current and past asthma among children, aged 13–14 years in Iqaluit (5.2% and 8.6%). As expected, this is lower than the prevalence of these conditions in the rest of Canada (18.2%, and 10.8%, respectively) [18] and the 16.8% reported current asthma among the Canadian youth (grades 6–10) [19]. Among adolescents aged 13–14 years, the prevalence rates of current symptoms of wheezes ranged from 13.7 to 33.0%, with a higher prevalence in Halifax and Saskatoon while lower in Vancouver [12]. Based on the initial ISAAC study, the lifetime prevalence of asthma among the children 13–14 years old was 19.2% in Hamilton and 12.2% in Saskatoon. The prevalence of wheezing in the 12 months before the survey was 30.6% in Hamilton and 24.0% in Saskatoon [20] and among children 6–14 years in rural Saskatchewan, Asthma prevalence was 14.7% [21]. The prevalence of asthma among children 5–19 years old (1996) in Prince Edward Island (17.9%), Halifax (17.1%), and Kingston (16.1%) was higher than that in Saskatoon (10.0%), Sherbrooke (9.7%) and Kelowna (11.9%). Among all 5–19 years old students, the prevalence of asthma was 13.0%, with the prevalence for males being higher than females [22].

Similarly, Chang et al. found that children with Inuit ancestry living outside Nunavut had a significantly lower prevalence of asthma than those with North American Indian and Métis ancestries being 5.7% for current asthma and 14.3% for past asthma [15]. The findings of this study are also in agreement with previously reported studies, where Aboriginal children (5.7%) had significantly lower levels of asthma prevalence than non-Aboriginal children (10.0%) in northern Canada [23]. Asthma has been reported as rare among the Inuit of Greenland [24] and low rate of Asthma (3.9%) was reported in Nikel, a Russian arctic city [25] while reported at a little bit higher prevalence of 11.9% in the Arctic region of Norway [26] and 12.3% in Northern Norway [8] and in Copenhagen [27]. In Sweden 2008, asthma among 15 year old adolescents was 14.3% [28].

There has been an increasing prevalence of asthma ever in Norway (7.3% in 1985 to 17.6% in 2008, p for trend < .001) [11] as well as in Canada (8.5% in 1996 to 13.3% in 2005) [29] emphasizing the need for follow up studies at a larger scale.

The prevalence of current and past allergic rhinitis are low at 8.6% and 10.3% respectively, This is lower than the reported 14.6 to 22.6% found among the same age group in five other southern Canadian cities [12] and much lower than that found in another 2 southern Canadian cities 45.8% in Hamilton and 33.8% in Saskatoon [20] also lower than reported in the arctic region of Norway at 26.0% [26] and the 32.8% in Denmark [30] and 26.4% in Sweden [28] but closer to the low rate of allergic rhinitis (13.9%) that was reported in Nikel, a Russian arctic city [25]. The average overall prevalence of current rhinoconjunctivitis symptoms worldwide was 14.6% for the 13- to 14-year old children (range 1.0–45) as found by Ait-Khaled et al. [31].

This difference is possibly related to less allergen exposure in Nunavut.

Interestingly, the prevalence of past allergic rhinitis among the non-Inuit was 16.7% while there were no cases of current allergic rhinitis. This could be attributed to transient symptoms when travelling outside Nunavut or being living outside Nunavut earlier in life because of an exposure to a triggering allergen that is absent or less abundant in Nunavut.

The current eczema prevalence amongst the grade seven students is 27.6%, with the highest prevalence recorded among the non-Inuit ethnicity at 50% but only 14.7% among the Inuit, with female predominance. Past eczema prevalence is 34.5%, with the highest prevalence among the non-Inuit at 66.7% while 17.6% among the Inuit which is comparable to that found among the Mushua Inuit of Natuashish (a minority of Inuit people living in Labrador, Canada) who had a prevalence of 16.5%, with female predominance as well [13]. In agreement with our study findings, Weiland et al. have previously reported that the prevalence of eczema symptoms correlated positively with latitude and negatively with the mean annual outdoor temperature [10], thus, making it an expected finding to get a higher eczema rate in Nunavut compared to that in southern Canada. Similarly, a high prevalence of eczema (23.6%) was reported in Arctic Norway [8] compared to a lower prevalence of 12.7% in the southern part of the same country [32, 33].

Interestingly, a more recent study showed a lower eczema prevalence of 10.4% in Arctic Norway [26], also lower rates of 8.1% and 12.3% were reported in Denmark [30] and 11.4% in Sweden [28]. Among adolescents aged 13–14 years, the prevalence rates of current symptoms of eczema in 5 southern Canadian cities were also lower than Iqaluit and ranged between 8.2 and 10.4% [12] also less than two other southern Canadian cities of Saskatoon and Hamilton at 17.3% and 14.5% respectively [20]. For the age group 13 to 14 years, data on 663,256 participants from 230 centers in 96 countries showed eczema prevalence values ranging from .2% in China to 24.6% in Columbia with the highest values in Africa and Latin America [6].

Contrary to our findings in the previous study among grade one students [1], we found a higher prevalence of eczema among the non-Inuit. Taking into consideration the low number of participants especially the non-Inuit, there could be a selection bias that most of those who participated were the ones with eczema.

The estimates of the annual costs of eczema in Canada is about $1.4 billion [34] which can impose a considerable financial burden on the lower socioeconomic populations in the far North.

Our study shows a surprisingly high prevalence of sensitization to cats, at 29.2%, which is much higher than the 8.6% prevalence among grade 1 to 8 children in the province of Saskatchewan [35] being much lower among the Inuit who tend to have a dog rather than a cat at home.

The prevalence of sensitization to trees is 8.3%, all of them among the mixed ethnicity (compromising 22.2% of that ethnicity), and the sensitization to grass at 20.8%, most of the cases were also among the mixed ethnicity might be related to the high rate of being ever outside Nunavut, knowing that no trees grow in Nunavut.

The absence of sensitization to house dust mite, mold, weed, ragweed and dog in our study is consistent with the findings in our previous study among grade one students [1] and in Greenland where the prevalence of sensitization to house dust mite is four times lower than that in Denmark, probably because of lower indoors house dust mite count [36], also in Northern Norway had similar findings with low house dust mite and mold sensitization [37]. Similarly, a study in Northern Sweden showed that sensitization to cats was the most common at 19% while sensitization to mites and mold was uncommon [38].

A previous house survey in Nunavut have shown that although building’s fungal concentrations were low, the mattress fungal levels were markedly increased, and the dust mites were virtually non-existent [39].

The strengths of this study include using a validated ISAAC questionnaire with the added relevant questions to this population and geographical area, also having a relatively high percentage of participation compared to previous studies in Nunavut.

It is a limitation that our study population is small and that 53.7% filled the questionnaire while only 22.2% attended the skin prick testing, this led to difficulty in getting a strong statistical power and caused wide confidence intervals which limits our ability to have solid conclusions and definitive statements. There is a risk for selection bias, taking into consideration that families with children having current atopic symptoms are more keen to participate in such a study and attend the skin prick testing, giving rise to a possible overestimation of the prevalences. The recall bias remains a possibility as with every retrospective questionnaire-based study. A larger scale longitudinal study is highly recommended to avoid such limitations.

Despite those limitations, we believe that our study contributes substantively to the existing literature because of the scarcity of data about allergies and allergic conditions among the Inuit population and other ethnicities living in Nunavut, serving as a benchmark for future prevalence studies.

## Conclusion

While being cautious about firm conclusions due to the small sample size and power, the noticed variations in the prevalence and risk factors of asthma, allergic rhinitis and eczema among different ethnicities living at the same subarctic environment might be related to several possible explanations like genetic, gene-environment interaction and/or lifestyle factors, it was out of the scope of this study to determine the causality of such variation in prevalence, which emphasizes the need for further investigation.

## Abbreviations

ISAAC: International Study of Allergy and Asthma in Children
SPT: skin prick test
sq. km: square kilometer
HDM: house dust mite
OR: odds ratio
CI-95: confidence interval of 95%
AR: allergic rhinitis
